# Digitalized Thermal Inspection Method of the Low-Frequency Stimulation Pads for Preventing Low-Temperature Burn in Sensitive Skin

**DOI:** 10.3390/bioengineering12060560

**Published:** 2025-05-23

**Authors:** HyungTae Kim, Jong-ik Song, Ji-won Seo, CheolWoong Ko, Gi-ho Seo, Sang Kuy Han

**Affiliations:** 1 Korea Institute of Industrial Technology, Ansan 15588, Republic of Korea; htkim@kitech.re.kr (H.K.); cheko@kitech.re.kr (C.K.); 2 TRYCAM Technology, Ansan 15495, Republic of Korea; jongik11@naver.com (J.-i.S.); duqrlehfl@naver.com (J.-w.S.);

**Keywords:** low frequency stimulation, low-temperature burn, thermal imaging, data digitalization, safety evaluation

## Abstract

An accurate thermal measurement of low-frequency stimulation (LFS) pads for thermotherapy was investigated using background subtraction (BGS) methods. The safety of LFS thermal pads must be investigated to prevent low-temperature burns (LTBs), because they frequently contact the sensitive skin in neck, shoulder and abdominal regions. The thermal measurement was based on thermal imaging using the active region-of-interest (ROI) from a foreground. The shape of the LFS thermal pad consists of complicated curves, thus it is difficult to extract the foreground using conventional shapes of ROIs. We proposed the foreground extraction using background subtraction (BGS) and digital and morphological filters to time-variant thermal images. The foreground extraction was implemented using open sources and experimented for abdominal, cervical and patellar pads. The results showed that the foreground can be separated from background regardless of the size, position, orientation and shape of the pad. The thermal characteristics of the LFS thermal pads were evaluated from the complicated shapes of the foreground with high accuracy. This study demonstrated that standard deviation of pixel history (SDPH) is a simple method for the BGS, but the SDPH is useful to find the safety risk of LTBs and prevent them in advance. The results also showed that the proposed SDPH was simple but had remarkable accuracy compared with the conventional BGS methods. These BGS methods are expected to increase the reliability of products used on the human body. Further, the BGS methods can be used to inspect the temperatures of static products in industrial processes.

## 1. Introduction

Low-frequency stimulation (LFS) is a non-invasive therapy where electric pulses applied to the skin are used to stimulate muscles [1]. Electric pulses used to apply LFS to reduce muscular pain are typically below 20 Hz and shaped by various signal patterns [2]. The LFS induces involuntary muscle contractions and reduces acute atrophy in response to electric pulses, thereby promoting muscle strength and recovery [3]. Further, LFS is effective in relieving chronic pain and fatigue, and therefore, it is widely used in treatment and rehabilitation of myalgia [4]. Experiments in previous studies have implied that LFS increases the oxidation potential of stimulated muscles and is a safe and well-tolerated therapy [5,6].

The electrode for the LFS has 2–5 poles and is installed on a silicon pad to maintain contact with the skin. The silicon pads are patched to the pain spots of the patients and wired to a controller before performing the LFS [7]. The controller pulses and repeats electric waves into the pain spots through the electrodes in the pads. Electro-thermal warmers are occasionally equipped with pads to improve efficacy and compliance. Thermotherapy increases blood flow in the muscle and is effective for muscle relaxation [8]. However, long exposure to low heat can cause low-temperature burns (LTBs) during thermotherapy even when using mild heat sources, and electric pads are the most common cause of this [9]. For children, the temperature is dropped by 3–4 °C to ensure protection from burns [10].

The LFS protocols for clinical use and typical applications suggest treatment times of 30 min. Some studies extend this to 60 min under test conditions [4], which can lead to LTBs. The number of patients with musculoskeletal disorders and muscle pain is expected to increase with the increase in the aging population and sporting activities. Moreover, pain in the cervical and shoulder joints has increased because of the excessive usage of smart devices [11]. Therefore, there is a stable demand for the LFS thermal devices, and household applications are expected to gain popularity. Recent studies presented LFS devices for use in daily life and as wearable devices [12,13]. However, a major concern regarding household distribution is the possible increase in LTBs from abuse or misuse cases. Therefore, the thermal characteristics of the LFS thermal devices need to be verified to prevent LTBs. Conventional LFS thermal devices target the abdomen, neck and knees, which have sensitive skin, and therefore, thermal characteristics have to be assessed for product safety. Thus, safety functions are recommended for commercial heating pads contacting skin to prevent LTBs from prolonged usage [9,14]. Safety functions have been achieved using timers and overheat shut-offs; however, they control the electric currents before the pads were using point-based temperature. A transparent heating pad was developed to monitor the skin color for LTB prevention [15], but its thermal characteristics must be verified before public release. Many studies have been proposing education and developing strategies for preventative measures of LTBs [16,17]. Thus, a thermographic method was employed to verify temperature distribution and thermal characteristics of the pads in this study.

An LFS thermal pad needs to exhibit characteristics such as stability, thermal uniformity and extreme temperature within its skin-contact area. A thermocouple, which is a conventional sensor used to measure temperature, is employed to verify the LFS device on the forearms in [18]. Although the thermocouple provides accurate and reliable temperature, the response is slow, and the measurement range is narrow. The thermocouple can measure the temperature at one spot, and therefore, extreme temperatures cannot reliably be detected over the area of the LFS thermal pad. A thermocouple is made of conductive materials that generate the thermoelectric effect, and therefore, the electric current during LFS can affect the measurement results. Unlike thermocouples, thermal imaging is a non-contact, non-destructive, rapid and intuitive method for measuring the temperature of an object [19]. Thermal imaging is a practical approach for monitoring the thermal characteristics of skin contact devices to prevent LTBs [20]. Thermal imaging and thermography can detect the optical responses of a long-wave infrared ray (LWIR), which has a wavelength range of 7–14 μm [21]. Thermal imaging has been widely used to observe human skin for dermatological diagnosis, pressure injury, arterial disease, pharmaceutical inspection and physiotherapy efficacy [22,23,24,25,26]. Further, it has also been used to investigate the effects of LFS on body fat [27]. Thermal imaging has been used to inspect warming devices that are in contact with human skin. A serpentine warmer attached to the skin on the forearm and temperature oscillations were investigated using thermal imaging in [28]. Thermal images were acquired during the operation of the graphene-based warmer to demonstrate the functionality of the capacitive temperature sensor patch in [29]. The operation of a thin, flexible and wireless patch applied across large areas of the skin in cooling and warming modes was verified using thermal imaging. The patch can interface with various body locations, such as the chest, back and abdomen, which have sensitive skin [30]. Therefore, thermal imaging is convenient to investigate the thermal characteristics of an LFS thermal pad.

Continuous and real-time monitoring of the LFS thermal pad is necessary for its safe use in clinics and homes. However, previous studies on thermal warmers on the skin used thermal images acquired at a specific time [28,29] did not analyze the thermal distribution, and did not investigate the statistical characteristics of the thermal device. Our pilot study discussed in the basis of active region-of-interest (ROI) to designate the measurement area and verified the accuracy of the ROI by visual examination [31]. This paper is an extended version of the pilot study and systematized the method. In this study, an inspection method was proposed to obtain the thermal characteristics from the inner region of the LFS thermal pad using background subtraction (BGS). BGS performs accurate feature detection of the LFS thermal pad and separates a ground truth for the temperature measurement. The ground truth was used for the ROI and a foreground mask to analyze the thermal characteristics. The ROI is generated from the deviation and morphology of the time-variant thermal images and the BGS methods. Using these approaches, the atypical shape of a target, i.e., the LFS thermal pad, can be separated from the background in sequential thermal images to ensure a greater accuracy of the thermal characteristics. The safety factors of the LTB are evaluated based on the thermal characteristics of the LFS thermal pad.

The remainder of this paper is organized as follows. In Section 2, we present the morphological background separation from the thermal images of the LFS thermal pad. In Section 3, we present results obtained using the ROI and detected features. In Section 4, we discuss the thermal characteristics and performance of the proposed method. Finally, in Section 5, we present the conclusions of this study.

## 2. Materials and Methods

### 2.1. Background Subtraction

**Figure 1:**
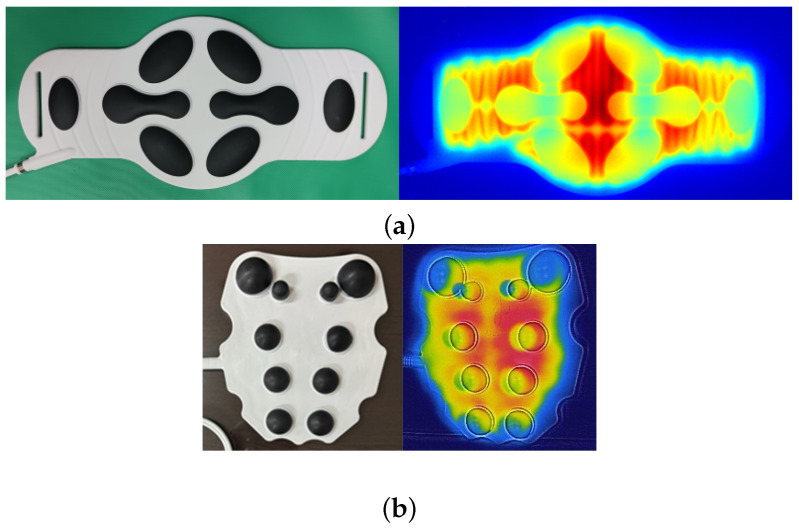
LFS thermal pads and their thermal images for (**a**) abdominal in lossless format and (**b**) cervical pain in loss compression format.

A thermal image includes target and background temperatures. Figure 1 shows thermal images of LFS thermal pads used for abdominal and cervical pain in rectangular ROIs [31]. The pads shown in the colored images are fastened to the painful area and warmed during LFS up to 60 min maximum. The blue area in the thermal images represents the background. The temperature of the LFS thermal pad varies according to the time and heat generation. The other colored areas indicate the pads and the temperature variations based on warming time. The internal circuits of the pads appear after full warming. The pads and background displayed in the thermal images exhibit complicated patterns, making it unfavorable to attempt geometric ROIs on background exclusion. The target in the thermal image can be extracted using a geometric ROI similar to that of the conventional machine vision systems. The rectangular ROIs include thermal images of the LFS thermal pads with a part of the background. Here, the background is redundant to the temperature measurement; however, the background is basically combined when using conventional geometric ROIs, and can affect the accuracy of the thermal measurement. The background temperature can cause a temperature error in the thermal analysis when image processing is applied. Thus, BGS has to be used to separate the background and foreground in a thermal image with a complicated shape.

Background temperature is lower than that of the target, and therefore, the ground truth of the target can be obtained by applying a temperature threshold. The foreground is used for various fields including medical diagnosis [32]. In Figure 1, the blue regions of the backgrounds indicate low ambient temperature, and thus, the backgrounds can be separated using a temperature threshold. However, temperatures of the ambient and LFS thermal pad vary according to the duration of use. The temperature threshold needs to be adjusted to separate the background in the time-variant thermal images. Although the foreground is crucial, there are no criteria for determining the temperature threshold. Thus, the ground truth is cropped manually by skilled personnel in many medical studies [32,33]. An automated process is required to observe the thermal characteristics of the LFS thermal pad, and therefore, manual cropping is impractical for thermal inspection. BGS was originally used to perceive moving subjects using a static camera, based on the static background hypothesis [34]. The basic principle involves evaluating the difference between the current image and reference image and updating the reference image according to the time variation.

(1) $$D_{t}\left(x,y\right)=\left|I_{t}\left(x,y\right)-B_{t}\left(x,y\right)\right|$$

where the $D_{t}$, *I*, *B*, *t*, and *x* and *y* represent the difference between frames, current image, reference image, time, and image coordinates, respectively. The variation in the foreground pixels is larger than that in the background pixels, and therefore, a foreground mask $M_{t}$ can be obtained by applying a threshold $\tau_{F}$.

(2) $$M_{t}\left(x,y\right)=\left\{\begin{array}{cc} 1 & \forall D_{t}\left(x,y\right)\geq \tau_{F}\\ 0 & \forall D_{t}\left(x,y\right)<\tau_{F} \end{array}\right.$$

The background mask is an inversion of the foreground mask. The background should be adjusted for changes in the scene over time; thus, a learning process must be conducted with a learning rate α based on the recursive approach.

(3) $$B_{t}\left(x,y\right)=\left(1-\alpha \right)B_{t-1}\left(x,y\right)+\alpha I_{t}\left(x,y\right)$$

Based on these principles, BGS has been developed into advanced algorithms such as count-based (CNT) [35], Godbehere–Matsukawa–Goldberg (GMG) [36], Google Summer of Code 2017 (GSOC) [37], local SVD binary pattern (LSBP) [38], mixture of Gaussian (MOG) [39] and K-nearest neighbor-based (KNN) [40]. BGS has been applied to detect moving objects; however, the LFS thermal pad is stationary during inspection. BGS can be employed for inspection because the static background and temperature vary in the foreground instead of moving. Thus, the foreground of the target can be achieved based on temperature variation. Further, the BGS provides atypical shapes of the foreground, and therefore, it is implemented in the experiment.

### 2.2. Deviation of Pixel History

These conventional BGS has been developed on the assumption of the movement of a small object in a field of view (FOV) and gradual change in illumination; thus the algorithms are complicated. The aim of BGS is to deduce the approximated silhouette and contours of a moving object. However, the inspection environment of the LFS thermal pad is static and a large object in a FOV. The accuracy of silhouette and temperature distribution are crucial for the inspection. Thus, a simple and definite algorithm based on the pixel variation in the image series was applied. Mean and standard deviation of image pixels were utilized for the BGS of consecutive static images [41]. Considering the inspection conditions, the background is invariant during inspection, whereas the LFS thermal pad is variant. Thus, the background can be separated using the variance or standard deviation among the consecutive thermal images. This approach was used for a robust feature detector for pedestrians in a thermal image and greater accuracy is therefore required [42]. Thus, in this paper, an accurate feature of a foreground can be achieved using this standard deviation of a pixel history (SDPH). The mean and the deviation images of sequential thermal images are simply obtained using the following equation [43]:

(4) $$\begin{array}{cc} {\bar{I}}_{t}\left(x,y\right)=\frac{1}{n}\sum_{i=t-n}^{t}I_{i}\left(x,y\right) & S_{t}^{2}\left(x,y\right)=\frac{1}{n}\sum_{i=t-n}^{t}I_{i}^{2}\left(x,y\right)-{\bar{I}}_{t}^{2}\left(x,y\right) \end{array}$$

where *n* is the number of thermal images selected in the sequence history of video frames before the current time. The environment of the temperature measurement is under limited and stable conditions, thus we propose to consider thermophysical properties for the foreground mask. The intensity and deviation of a foreground are higher than those of a background in this case of temperature measurement, thus the foreground mask can then be produced using the logical intersection of the thresholded images.

(5) $$M_{t}\left(x,y\right)=\left[{\bar{I}}_{t}\left(x,y\right)\geq \tau_{\bar{I}}\right]\cap \left[S_{t}\left(x,y\right)\geq \tau_{S}\right]$$

Equation (5) is intuitive, thus formulation is modifiable considering the thermophysical properties. For instance, a foreground of a cooling pad thresholds high temperature with an inverse inequation. The foreground is the measurement area, then it is obtained from the logical AND operation using the foreground mask.

(6) $$F_{t}\left(x,y\right)=M_{t}\left(x,y\right)\wedge I_{t}\left(x,y\right)$$

The learning process in Equation (3) is applicable if the changes in the background over time are significant.

### 2.3. Morphological Filtering

**Figure 2:**
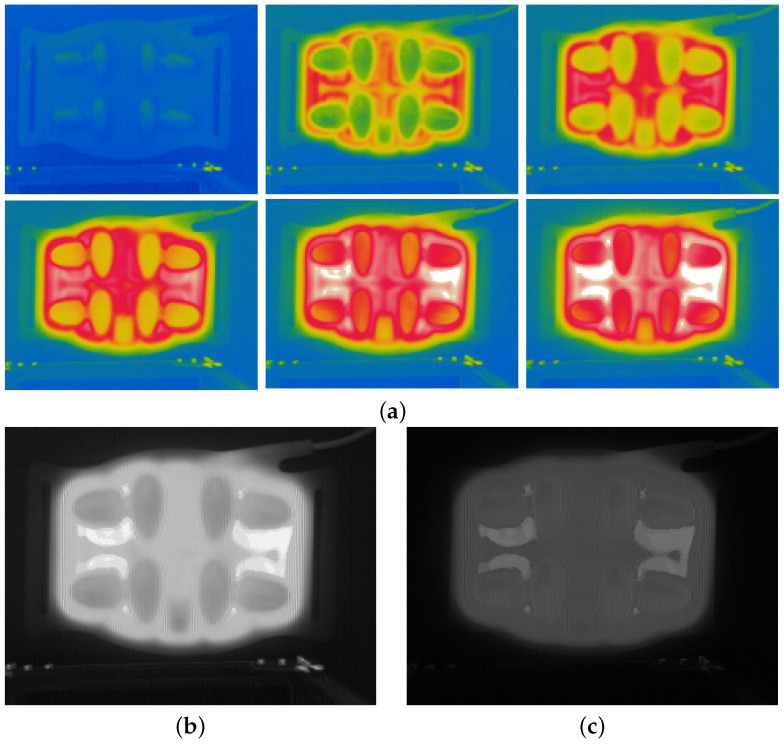

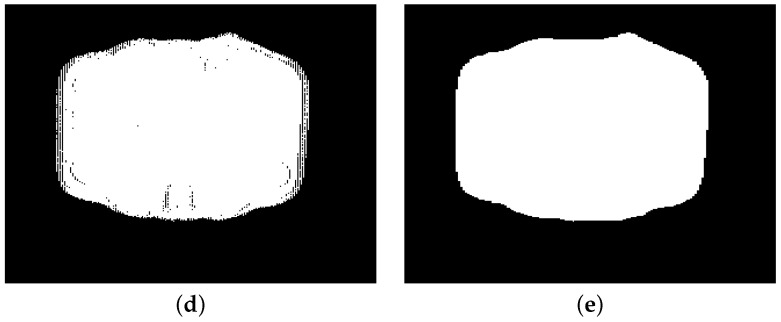
Example of thermal image processing: (**a**) color map of thermal images during warming of an abdominal pad; (**b**) average image; (**c**) deviation image; (**d**) raw foreground mask and (**e**) foreground mask after morphological filtering.

Figure 2 shows the image variations obtained using the proposed methods. Figure 2a shows the color maps of the thermal images according to warming time. The 13 images of the abdominal pad are acquired every 5 min for 60 min. The averages and deviations of the images before binarization are shown in Figure 2b and Figure 2c, respectively. Figure 2d shows the foreground mask after the logical operations in Equations (5) and (6). The boundary of the foreground mask is rough, and some inner holes are formed. The holes are discontinuous and scattered, and therefore, ordinary gap-filling methods are adverse. The foreground mask can be remedied using morphological filters to eliminate holes [44]. The foreground mask for stationary objects may not sufficiently encompass the entire foreground area; therefore, this can be solved by dilating the foreground mask [45]. Dilation was used to expand the boundary and shrink the inner holes. The dilation of the foreground mask is expressed as follows:

(7) $$M_{t}^{\prime }=M_{t}\left(x,y\right)⊕E=\left\{z|[{\left(\hat{E}\right)}_{z}\cap A]\neq \emptyset \right\}$$

where $\hat{E}$ indicates the reflection set of a structuring element, *E*, and *z* represents the translational displacement at $M_{t}$. *E* is a square-sized operator filled with 0 s and 1 s. The dilation of $M_{t}$ is the set of all displacements *z* such that the elements of $\hat{E}$ overlap with at least one element of $M_{t}$ [46]. The dilation inflates the foreground but also expands the dots and speckle patterns in the background. Erosion is the inverse of dilation and is a morphological operation that contracts bright pixels at the boundary, shrinking the object. Thus, erosion can restore the original foreground shape and eliminate the background noise patterns. Erosion is defined as forms similar to dilation.

(8) $$M_{t}^{\prime \prime }=M_{t}^{\prime }\left(x,y\right)⊖E=\left\{z|[{\left(E\right)}_{z}\cap A^{c}]\neq \emptyset \right\}$$

Equation (8) indicates that *E* must be contained in $M_{t}^{\prime }$ after being translated by all displacements *z*. In other words, *E* does not share any common elements with the background [46]. Combination filtering using dilation and erosion exhibited good performances in extracting the ground truth [47]. Dilation was applied to recover the original size after the erosion.

(9) $$M_{t}^{\prime \prime \prime }=M_{t}^{\prime \prime }\left(x,y\right)⊕E$$

This morphological filtering is iterated to remove holes and noise patterns sufficiently. The iteration numbers of the dilations must be equal to that of the erosion. Then, the foreground can be cropped by applying the morphologic mask $M_{t}^{\prime \prime \prime }$, as indicated in Figure 2e, to Equation (6).

### 2.4. Thermophysical Conditions

Extreme temperatures, thermal uniformity and stability are required to evaluate the safety risks of LTBs. Extreme temperatures are determined by searching for the minimum and maximum temperatures in the foreground.

(10) $$\left(T_{min},T_{max}\right)=\left[\arg\min_{x,y\in F_{t}}F_{t}\left(x,y\right),\arg\max_{x,y\in F_{t}}F_{t}\left(x,y\right)\right]$$

Thermal uniformity was obtained from the thermal distribution in the foreground. For example, the mean and standard deviation of the temperature in the foreground is obtained from the pixels in $F_{t}\left(x,y\right)$.

(11) $$\left(\mu_{t},\sigma_{t}^{2}\right)=\left[\frac{1}{n^{\prime }}\sum_{x,y\in F_{t}}^{n^{\prime }}F_{t}\left(x,y\right),\sum_{x,y\in F_{t}}^{n^{\prime }}F_{t}^{2}\left(x,y\right)-\mu_{t}^{2}\right]$$

Another characteristic can be obtained from the histogram of the thermal image h. A histogram counts the number of pixels classified by intensity, and the temperature at the maximum count number indicates the dominant temperature. A pixel ratio above the threshold temperature represents an area with full warming.

(12) $$\left(T_{dom},A_{h}\right)=\left[\arg\max_{z\in F_{t}}h\left(z\right),\frac{1}{n^{\prime }}\sum_{z\in F_{t}}h\left(z\geq \tau_{h}\right)\right]$$

The foreground functions as an active ROI, which provides signals and information from the thermal images. The thermal stability is a time-variant property, and therefore, it can be achieved by monitoring $T_{min}$, $T_{max}$, $T_{dom}$, $\mu_{t}$, $\sigma_{t}^{2}$ and *h*. Transient and steady-state responses in electric and control engineering can be adopted to depict thermal stability using these properties.

### 2.5. Experiment

**Figure 3:**
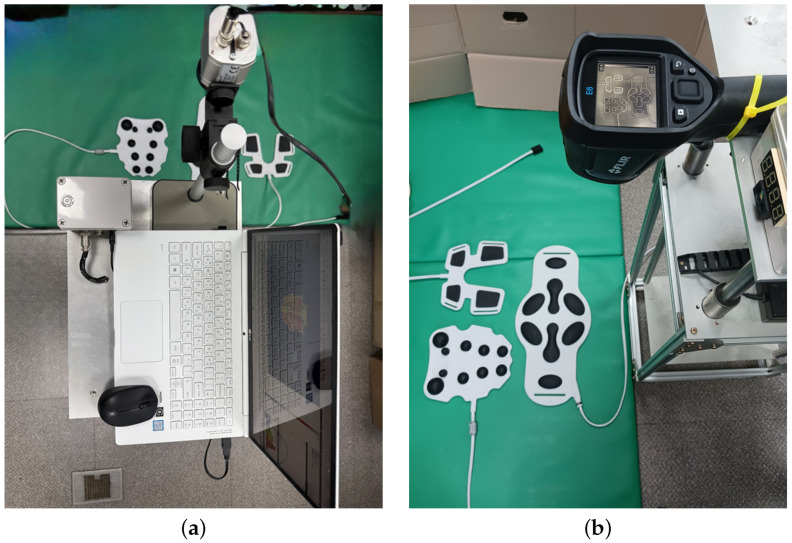
Experimental setups: (**a**) Optris PI640 and (**b**) FLIR E6-XT.

As shown in Figure 3, two types of thermal cameras were used to acquire the thermal images of the LFS thermal pads. The inspection targets included the abdominal, cervical and patellar pads. Thermal images in the lossless format TIFF were observed using an PI640 camera (Optris, Berlin, Germany) with a pixel resolution of 640 × 480, 10-bit pixel depth and temperature accuracy of 75 mK. The other camera, E6-XT (FLIR, Wilsonville, OR, USA), captured thermal images in the 8-bit loss compression format, JPG, with a pixel resolution 320 × 240 and a temperature accuracy of 60 mK. These industrial cameras are reliable for their accuracy and durability over long periods in manufacturing environments. The pads were placed on the floor and a thermal camera was installed on the vertical frame. Thermal images were obtained every 5 min from pads for 1 h. The time internal was determined after considering the slow responses of the pads. The thermal images were captured in an experiment room, under LED illumination with air conditioning that was isolated from other rooms. The thermal images were acquired in gray-scale with a fixed range between 25 °C and 45 °C. The proposed methods and conventional BGS were executed in C++ 17 code using open sources. The development platform was OpenCV 4.8.0 and operating system was Ubuntu Linux 20.04. The matrix operations of the thermal images were handled using OpenCV matrix, and the SDPH was achieved combining cv::multiply and cv::threshold. Variation was used instead of standard deviation because of the low sensitivity of SDPH in the experiment. The cv::bitwise_and() function was used for the intersection operation for the foreground mask. The cv::dilate() and cv::erode() functions were applied to dilation and erosion operations, respectively, with the iteration factors.

The experiment was conducted using conventional BGS methods such as CNT, GMG, GSOC, LSBP, MOG and KNN. These methods were implemented using cv::bgsegm provided by OpenCV. Dilation–erosion–dilation was performed to fill the holes and remove noise factors in the foreground mask. There were three and six repetitions of dilation and erosion, respectively. The dilation fills in the foreground, and double erosion shapes the dilated features to be smaller than the original size. Subsequently, the original size was restored during the final dilation. The parameters of the conventional methods were determined when holes and noises were minimized in the foreground mask. In total, 3–10 images were selected as the history factors for the above methods. The algorithm parameters of the BGS methods were mostly dependent on the threshold and number of images for the pixel history in this case; thus, the default values of OpenCV BGS were applied, except for the threshold. An edge detector, flood fill algorithm and manual correction were performed on the thermal images at *t* = 50 min when generating the ideal foreground masks of the LFS thermal pads. Template matching was applied to evaluate the difference between the ideal masks and masks generated by BGS methods. A hexa-core AMD Ryzen 3950X processor with 64 GB of memory was used to test the methods.

## 3. Results

Table 1 lists the foreground masks of the abdominal, cervical and patellar pads processed in a lossless image format. The two rows at the top of Table 1 show photos and ideal masks of the LFS thermal pads. The third row shows the thermal images acquired at *t* = 50 min and the next row shows the full-size image after processing the BGS. The foreground masks were sequentially varied according to warming time, and therefore, the foreground mask for measurement was selected from the time sequence of the masks. The matching coefficients between the ideal and the obtained masks are shown in the mask images. Foreground masks should ideally be shaped as closed curved areas without inner holes. The backgrounds were removed well; however, some dots and blobs in the background were observed when using LSPB and MOG. The thermal energy spreads along the pads after a sufficient amount of time has passed, and the shapes of the obtained masks become similar to the original shapes of the pads. The matching coefficients in Table 1 are greater than 0.7, indicating the similarity of the shapes of the ideal and obtained masks. GSOC showed the highest matching coefficient in the abdominal pad, and that of SDPH (*n* = 3) was slightly lower than that of GSOC. The matching coefficient of the KNN method was the largest for the cervical and patellar pads, followed by that of SDPH. The foreground masks created by GSOC, KNN and SDPH formed shapes that were more similar to those of the ideal mask compared to those of the others. The last row shows the color-mapped foregrounds obtained using the SDPH with a sufficient number of images of history pixels (*n* = 10) after 50 min. The color changed from blue to red with an increase in temperature. The matching coefficients increased compared with a smaller number of images for pixel history (*n* = 3). Thus, the thermal distribution, excluding the background, can be observed after applying the BGS methods and the SDPH provides relatively accurate shapes.

Table 2 lists the foreground masks of the same pads obtained in the loss compression format. The thermal image also included marks, characters and a scale bar in the corners; however, these were clearly removed after processing the BGS. The obtained masks formed a closed curved area without an inner hole; however, internal holes were observed in the case of the MOG. Noise patterns were observed in the images in the loss compression format, although BGS performed well. Foreground masks of the abdominal and patellar pads were well generated by applying the BGS. In the case of the cervical pad, GSOC, LSBP, KNN and SDPH provided shapes similar to the actual pad. The SDPH showed the highest similarity to the ideal mask of the abdominal and cervical pads, considering the matching coefficients. The similarity of the foreground masks in the patellar pad showed few differences based on the BGS methods.

## 4. Discussion

**Figure 4:**
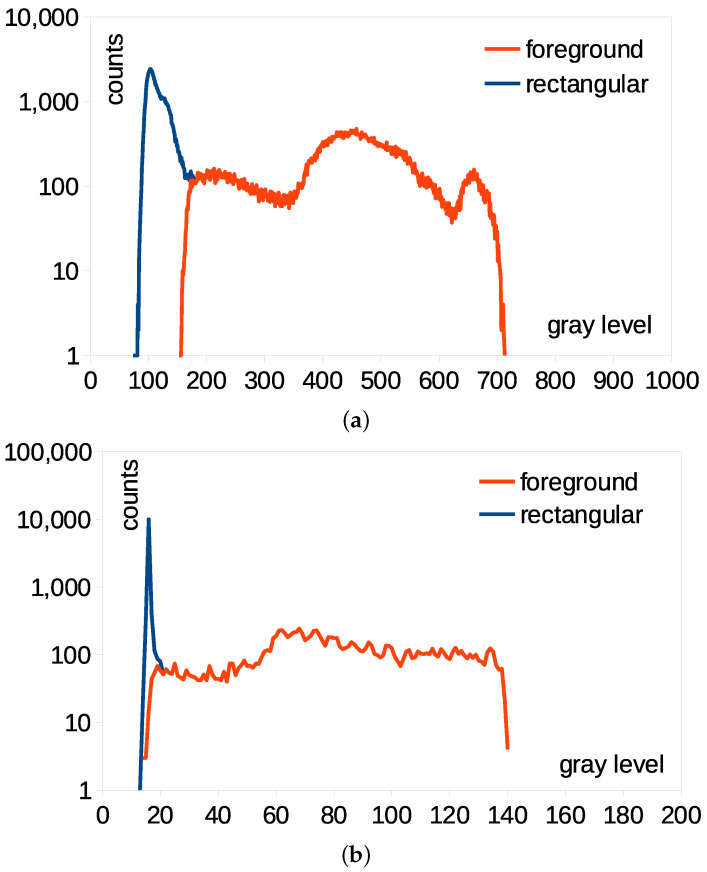
Image histogram comparison between the foreground and rectangular ROI for the abdominal pad using (**a**) lossless image and (**b**) loss compression formats.

The foreground generated by LSBP and MOG showed coarse boundaries, and SDPH produced a comparatively accurate foreground mask. CNT, GSOC, GMG and KNN were available for foreground extraction when inspecting LFS thermal pads. Thus, they can complement the SDPH in the foreground detection of thermal inspection from image sequences. The foreground mask obtained by SDPH better matched with the original shapes regardless of the shape, size and position. The processing time was lass than 30 ms in the worst-case scenario, and therefore, the computational cost was low. The BGS effect is noticeable in the image histograms shown in Figure 4 that were obtained using a foreground mask and a conventional rectangular ROI using the SDPH for the abdominal pad. The horizontal and vertical axes represent the gray level and pixel counts in the log scale, respectively. In both image formats, the pixel counts of the higher gray levels were equal; however, those of the lower gray levels were larger in the histogram of the rectangular ROI. The background relates the low gray level, and therefore, the SDPH eliminated the background effect. The maximum pixel counts of the rectangular ROI were placed at a lower gray level, and therefore, the background included in the conventional ROI significantly affected the temperature measurement. The shape of the histogram after SDPH became uniform was comparable to that of the conventional ROI.

**Figure 5:**
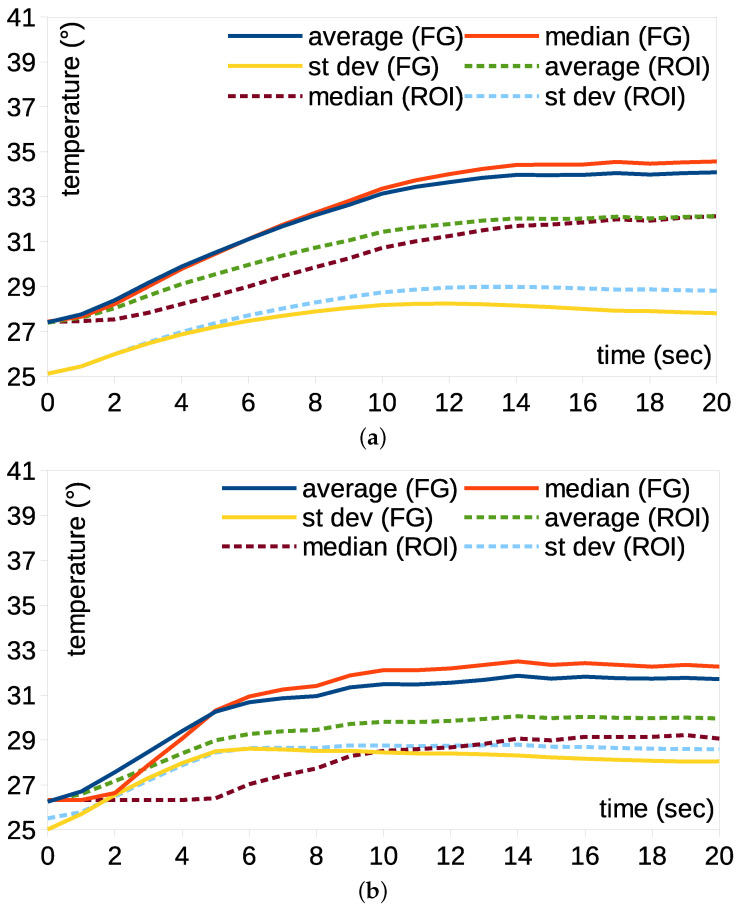
Temperature-variant comparison between foreground and rectangular ROIs for the cervical pad using (**a**) lossless image and (**b**) loss compression formats.

Temperature variation can be expressed using a statistical analysis of the pixels in the foreground mask or ROI. Figure 5 shows the variation in the average, median and deviation of the temperature with time. The dotted lines are the values from the rectangular ROIs and the continuous lines indicate the values from the foreground. The rectangular ROIs were manually placed to wrap the area of neck pads. The average and median temperatures increased when warming began, and they became saturated over time. The average and median foreground temperatures were higher than those of the rectangular ROIs. The average and median values of the foreground were similar; however, those of the rectangular ROIs were comparatively large. This implies that the background temperature decreased the measured values. The standard deviation of the foreground was lower, suggesting that the temperature distribution was uniform because of the background exclusion.

**Figure 6:**
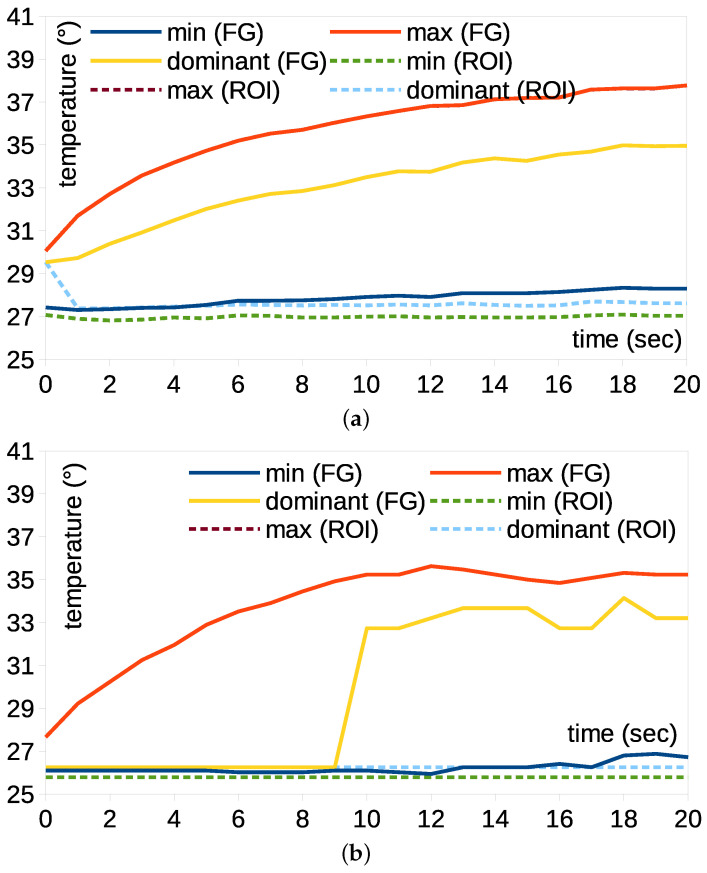
Extreme temperature comparison between the foreground and rectangular ROI for the patellar pad using (**a**) lossless image and (**b**) loss compression formats.

Figure 6 shows the variations in the minimum, maximum and dominant temperatures of the knee pad. The maximum temperatures of the foreground and rectangular ROI were equal because they are related to warming. However, the minimum and dominant temperatures of the foreground increased gradually, whereas those of the rectangular ROI remained invariant. This indicates that the background temperature was dominant in the rectangular ROI. However, the foreground detected the expansion of thermal energy to the boundary of the knee pad, which excludes the background. Further, this implies that the measurement accuracy of temperature became higher because the background effect became lower. Therefore, the accuracy of thermal monitoring for LFS thermal pads can be improved using BGS and foreground extraction. These accurate foreground data are useful for verifying the LFS thermal pads and preventing LTBs in advance.

BGS is applicable to industrial thermal monitoring. Conventional BGS methods aim to detect movements in a video, whereas this study detects intensity variations in the static foreground. Many factories monitor temperature variations in spaces, products and facilities during their processes. Based on the temperature measurement in a furnace, a thermal image of the molten steel was acquired in the fixed condition of the furnace and thermal camera. The temperature variation in the molten steel has been recorded in image volume, that is, accumulation of the image sequences. SDPH detects the foreground from the temperature variation in the accumulated images based on pixels, and therefore, it is advantageous for measurement of a static object and inspection of a process.

Based on these facts, a foreground is desirable for temperature measurement and improves measurement accuracy when using a thermal camera. The SDPH is easy to implement and consumes a low processing cost, and therefore, it is expected to be useful in thermal imaging devices. The transmittance of LWIR in solid materials is superior to that of visible rays, and thus, thermal inspection with a foreground is applicable to thermal inspections in diverse devices including semiconductors, secondary cells, flat panel displays as well as medical devices. The SDPH and BGS methods are complementary for collecting training data for artificial intelligence from big data mining. We plan to apply the SDPH and BGS to the analysis of fluid flow behavior on porous substrates. However, the foreground mask was manually selected from a candidate group in the time sequence, and we will discuss automatic selection of a foreground mask in the next study. This automatic function will increase convenience of thermal measurement devices.

## 5. Conclusions

Foreground extraction from a thermal image sequence was proposed to inspect the characteristics of LFS thermal pads and prevent LTBs. SDPH and BGS methods were applied to generate foreground masks of LFS thermal pads using image volumes. Defective and noisy patterns in the foreground mask and background were eliminated using morphological operations, and then, the foreground was extracted from the foreground mask and thermophysical conditions. In most cases, the SDPH precisely extracts a foreground from thermal images in static conditions and thermal characteristics of a complicated shape can be obtained from the foreground with high accuracy. The extreme temperatures and thermal uniformity of the foreground were obtained by excluding the background. The thermal characteristics using SDPH presented higher accuracy; thus, SDPH is beneficial to the thermal measurement of a static object. The SDPH is simple and easy for implement, and it is expected to contribute to improving the accuracy and convenience of thermal imaging in the industry. The computational cost and algorithm complexity of SDPH are low, and therefore, SDPH is applicable to portable devices of thermal imaging. The SDPH and BGS methods accurately crop target images, thus they will be useful to collect training images and mine big data for artificial intelligence. The foreground mask was manually determined in time sequence, thus we will present an automatic selection method for thermal measurement devices in the next study.

## Figures and Tables

**Table 1:** Masks, template matching and foregrounds of lossless image format using BGS methods.

Type	Abdominal Pad	Cervical Pad 2	Patellar Pad
Photo	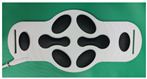	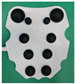	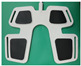
Ideal	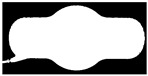	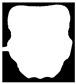	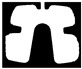
LWIR ( *t* = 50 min)	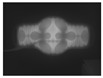	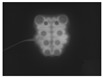	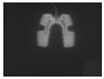
CNT	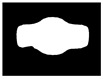 0.823	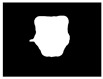 0.892	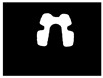 0.745
GMG	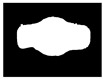 0.845	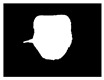 0.796	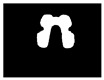 0.733
GSOC	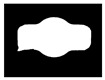 0.880	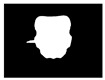 0.971	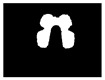 0.803
LSBP	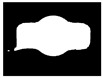 0.877	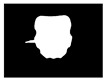 0.947	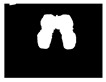 0.791
MOG	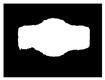 0.825	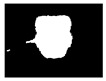 0.928	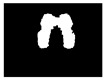 0.739
KNN	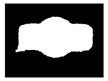 0.868	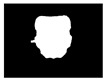 0.975	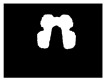 0.823
SDPH ( *n* = 3)	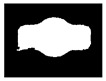 0.865	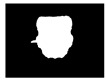 0.972	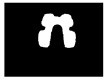 0.805
SDPH ( *n* = 10)	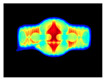 0.887	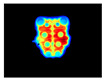 0.977	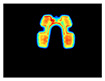 0.819

**Table 2:** Masks, template matching and foregrounds of loss compression format using BGS methods.

Type	Abdominal Pad	Cervical Pad 2	Patellar Pad
Photo	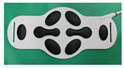	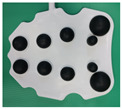	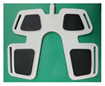
Ideal	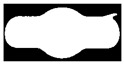	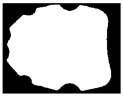	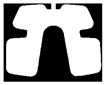
LWIR ( *t* = 50 min)	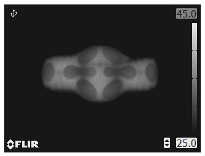	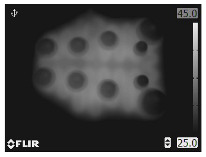	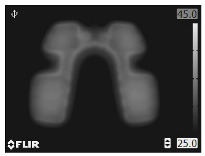
CNT	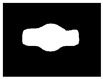 0.716	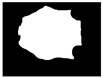 0.685	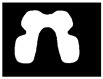 0.768
GMG	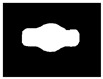 0.753	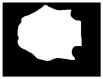 0.715	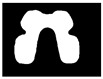 0.784
GSOC	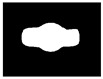 0.765	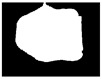 0.851	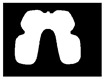 0.766
LSBP	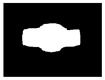 0.778	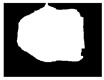 0.844	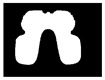 0.771
MOG	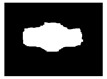 0.797	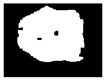 0.807	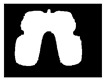 0.745
KNN	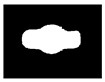 0.782	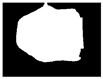 0.843	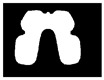 0.773
SDPH ( *n* = 3)	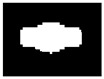 0.834	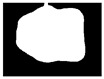 0.895	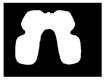 0.773
SDPH ( *n* = 10)	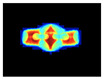 0.821	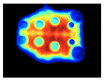 0.900	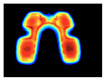 0.777

## Data Availability

The original contributions presented in this study are included in the article. Further inquiries can be directed to the corresponding author.

## Abbreviations

The following abbreviations are used in this manuscript:

CNT	Count-based
FOV	Field of view
GMG	Godbehere–Matsukawa–Goldberg
GSOC	Google Summer of Code 2017
KNN	K–nearest neighbor
LFS	Low frequency stimulation
LSBP	Local SVD binary pattern
LTB	Low-temperature burn
LWIR	Long-wave infrared ray
MOG	Mixture of Gaussian
ROI	Region of interest
SDPH	Standard deviation of a pixel history
